# Editorial: The interplay between the oral Microbiota and rheumatoid arthritis

**DOI:** 10.3389/froh.2022.1055482

**Published:** 2022-11-08

**Authors:** Daniel Manoil, Nagihan Bostanci, Axel Finckh

**Affiliations:** ^1^ Division of Cariology and Endodontics, University Clinics of Dental Medicine, Faculty of Medicine, University of Geneva, Geneva, Switzerland; ^2^ Section of Cariology and Endodontics, Division of Oral Diseases, Department of Dental Medicine, Karolinska Institutet, Stockholm, Sweden; ^3^ Section of Oral Health and Periodontology, Division of Oral Diseases, Department of Dental Medicine, Karolinska Institutet, Stockholm, Sweden; ^4^ Division of Rheumatology, Department of Internal Medicine Specialties, University Hospitals of Geneva, Geneva, Switzerland

**Keywords:** rheumatoid arthritis, risk factors, oral microbiota, periodontal inflammation, autoantibodies, anticitrullinated protein antibodies (ACPA), *Staphylococcus aureus* bacteraemia, *Porphyromonas ginigivalis*

**Editorial on the Research Topic** The interplay between the oral microbiota and rheumatoid arthritis

During the past 15 years, growing evidence has emerged to support associations between a dysbiotic oral ecosystem and the immune onset of rheumatoid arthritis (RA) (1). The articles included within this research topic endeavour to provide novel insights into the potentially causal associations between the oral microbiota and RA development.

RA is the most common inflammatory autoimmune disease. Its pathogenesis is characterized by the production of anticitrullinated proteins autoantibodies (ACPA), synovial inflammation and hyperplasia, leading to chronic erosive polyarthritis (2). Whereas RA exhibits approximately 1% prevalence in the general population, it is estimated that the genetic background accounts for up to 60% of the risk of developing the disease (3). As a corollary, first-degree relatives (FDRs) of patients with RA bear a 3 to 5-fold increased risk of developing the disease themselves, a risk that grows even higher in families with multiple cases (4). Despite a susceptible genetic make-up, the development of the disease requires interactions with environmental factors. Lately, considerable efforts attempted to determine if, and to what extent, periodontitis may represent one such environmental factor (5). While epidemiological, translational and mechanistic reports tend to support such an association (6–9), diverging data also exist (10), and the degree to which periodontitis may contribute to the aetiology of RA is yet-to-be fully understood. Interestingly, there is also evidence showing an enrichment of inflammophilic oral taxa in RA patients devoid of periodontal symptoms (11). Further worth mentioning is that the selection of patients, and knowledge of their potential medication is crucial to the validity of those observations, since the intake of anti-inflammatory and anti-rheumatic drugs has been shown to impact both the periodontal inflammatory response and the microbiota composition (12). In this research topic, Zekeridou et al. presents a synthetic overview of the current body of literature, with a particular focus on FDRs. Remarkably, there is evidence showing that serum antibodies against periodontal pathogens, such as *Porphyromonas gingivalis*, significantly associate with ACPA seropositivity (13), and sometimes to an even greater extent than long-established associations, such as those between RA and smoking (14). The authors discuss how FDRs may represent an ideal target population for primary and secondary preventive measures, given the important hereditability of RA (15). Indeed evidence suggests that the early diagnosis of RA-related autoimmunity, and its early treatment, could avert further development of the disease (16–18). If proven, a causal link between periodontitis and RA may therefore open the door to the identification of microbial profiles prone to cause dysbiosis and immune dysregulation, with promising public health perspectives for at-risk individuals (19). However, such an approach requires the comprehensive understanding of interactions between the microbiota and mucosal immunity, along with the accurate identification of biomarkers for the diagnosis of pre-symptomatic RA.

The identification of predictive biomarkers depends on a comprehensive understanding of the earliest immunological abnormalities and of the early environmental drivers of disease. One hallmark of early RA autoimmunity is the occurrence of serum ACPAs as early as 10 years prior to the first signs of joint involvement (20, 21). The detection of ACPAs in individuals devoid of articular symptoms suggests that these autoantibodies originate at extra-articular sites, and mucosal surfaces affected by an overlying dysbiosis were specifically incriminated. This is referred to as the “mucosal origins hypothesis” (22). In the case of periodontitis, this model is particularly supported by the finding that several periodontal pathogens can either citrullinated bacterial and host peptides as in the case of *Porphyromonas gingivalis*, or cause leukocyte hypercitrullination as in the case of *Aggregatibacter actinomycetemcomitans*, and thereby contribute to initiating an immune response against citrullinated epitopes. With this consideration, de Smit et al. investigated whether locally produced ACPAs (IgA isotype) detected in the gingival crevicular fluid of healthy individuals are associated with particular subgingival microbial profiles. In this timely original paper, participants were stratified into groups with or without periodontitis, and further into individuals displaying low (≤0.1 U/ml) or high (>0.1 U/ml) IgA-ACPA in their crevicular fluid. Data showed that periodontally affected individuals display a differentially abundant subgingival microbiota compared to periodontally healthy individuals. More importantly, the periodontally affected group displayed differences in taxonomic composition between low- and high-ACPA individuals, the latter exhibiting increased abundances of the family and genera Neisseriaceae, *Tannerella*, and *Haemophilus*. Furthermore, microbial differences between low- and high-ACPA individuals also emerged among periodontally healthy individuals. Specifically, increased abundances of *P. gingivalis* were characteristic of high-ACPAs individuals, thereby supporting an association between this periodontopathogen and a local ACPA response. While these data do not establish a causal association between periodontitis and RA etiopathogenesis, they unarguably support a contribution of periodontal dysbiosis to the local generation of IgA-ACPAs.

Beyond articular damage, established RA also substantially increases the risk of systemic complications, such as lymphomas, myocardial infarctions, interstitial fibrosis or susceptibility to infections. Specifically, recent evidence indicates that RA patients display and increased risk of *Staphylococcus aureus* bacteraemia (23, 24). This increased risk may be partially explained by an imbalance in inflammatory pathways during RA pathogenesis, and the use of antirheumatic treatments, that together impair immunity. In this research topic, du Teil Espina et al. suggests that *P. gingivalis* may also contribute to a higher risk of staphylococcal bacteraemia. This novel mechanistic paper shows that outer-membrane vesicles of *P. gingivalis* promote aggregation of *S. aureus* cells, and that this aggregation appears fostered by the presence of gingipains and the bacterial peptidylarginine deiminase (PPAD). Furthermore, the authors elegantly employ confocal microscopy and flow cytometry to show that outer-membrane vesicles of *P. gingivalis* also promote *S. aureus* internalisation within neutrophils. They postulate that such mechanism could turn neutrophils into “trojan horses” that help *S. aureus* translocate into the bloodstream. The authors, however, acknowledge a series of missing links to solidly establish a role of *P. gingivalis* in the risk of staphylococcal bacteraemia in RA, and these include a definite association between RA and *P. gingivalis*, proof of ecological co-localisation between *P. gingivalis* and *S. aureus*, and finally, evidence of *S. aureus* survival within neutrophils.

We are hopeful that this research topic may provide novel perspectives into the role that oral dysbiosis may play in the immune onset of RA, and highlight future developments required to understand the cross-talk between the human microbiota and immunity at mucosal surfaces.
